# Hepatitis C Management in a Lung Cancer Patient on Checkpoint Inhibition: A Case Report

**DOI:** 10.7759/cureus.102193

**Published:** 2026-01-24

**Authors:** Diana Póvoas, Patricia Garrido, Maria Francisca Moraes-Fontes, Maria José Manata, Fernando Maltez

**Affiliations:** 1 Infectious Diseases, Unidade Local de Saúde São José, Lisbon, PRT; 2 Lymphocyte Physiology, Gulbenkian Institute for Molecular Medicine, Oeiras, PRT; 3 Lung Unit, Champalimaud Foundation, Lisbon, PRT; 4 Immuno-Oncology Consultation, Champalimaud Foundation, Lisbon, PRT; 5 Infectious Diseases, Instituto de Saúde Ambiental, Faculdade de Medicina da Universidade de Lisboa, Lisbon, PRT

**Keywords:** checkpoint inhibitor therapy, chemotherapy treatment, directly-acting antiviral agents, hepatitis c (hcv) infection, non small cell lung cancer

## Abstract

Direct-acting antivirals (DAAs) have fundamentally changed the paradigm of chronic hepatitis C treatment, and their use is now recommended for all patients with active infection. Immunosuppression is a known contributing factor to an increased risk of disease progression, necroinflammatory activity, and chronic liver disease in individuals with chronic viral hepatitis, including both hepatitis B and C. Viral reactivation or flares of disease activity have been described in patients with chronic hepatitis B or C, respectively, who undergo immunosuppressive or immunomodulatory treatments, including chemotherapy and immunotherapy with checkpoint inhibitors. However, there is a paucity of guidance regarding the treatment of chronic hepatitis C in patients with cancer, and this population has traditionally been excluded from clinical trials. We report the clinical case of a 56-year-old male undergoing chemotherapy and immunotherapy for stage IVA lung adenocarcinoma and the successful treatment of a newly diagnosed hepatitis C infection, which was identified immediately before the onset of cancer treatment. We also provide a review of key aspects of hepatitis C screening and recent advances in hepatitis C virus (HCV) management among cancer patients.

## Introduction

Direct-acting antivirals (DAAs) inhibit nonstructural (NS) proteins, including NS3/4A, NS5A, and NS5B, which are essential for hepatitis C virus (HCV) replication. Among individuals with HCV infection, contemporary DAA regimens have demonstrated very high effectiveness, with sustained virological response (SVR) rates of ≥95% across HCV genotypes, excellent safety and tolerability, short treatment durations of 8-12 weeks, and oral administration with once-daily dosing [1]. Two pan-genotypic, fixed-dose combination regimens containing glecaprevir/pibrentasvir (G/P) or sofosbuvir/velpatasvir are now recommended as first-line therapies for chronic HCV infection for eight or 12 weeks, respectively [1].

Recently, immune checkpoint inhibitors (ICIs), including antibodies directed against programmed cell death 1 (PD-1) and its ligand, programmed death-ligand 1 (PD-L1), have revolutionized cancer treatment. ICIs are now approved for many types of cancer, particularly as standard first-line treatment in combination with chemotherapy for stage IV non-small cell lung cancer without a clinically actionable biomarker, especially in adenocarcinoma [2]. In addition to the toxicities of cytotoxic chemotherapy, patients may also experience ICI-associated immune-related adverse events (irAEs), which can involve the liver. Specifically, pembrolizumab therapy, an anti-PD-1 antibody, used for metastatic lung cancer, can be complicated by hepatic immune-related adverse events of grade 3 or higher, which have been reported in approximately 1% of patients when used in combination with chemotherapy.

Chemotherapy alone may also cause elevations in serum liver enzymes that are usually mild, transient, and self-limited in nature, and only rarely require dose modification or discontinuation. While ICI-induced hepatitis has been observationally associated with improved survival, in keeping with reports of greater ICI efficacy among patients who develop irAEs [3], clinically meaningful liver enzyme elevations may nonetheless compel treatment delays, interruptions, or even discontinuation of oncologic chemo/immunotherapy, which can be detrimental [4,5].

Cancer patients with chronic viral hepatitis face the risk of virological events, which vary by virus and serologic infection markers. HBV may reactivate during chemotherapy or ICI treatment in both HBsAg-positive and HBsAg-negative/HBc-positive patients, with the highest risk observed in HBsAg-positive individuals [6]. In chronic hepatitis C, we agree that the term “reactivation” should be avoided: in RNA-positive chronic HCV, a ≥1 log10 increase in RNA levels accompanied by alanine aminotransferase (ALT) elevation represents an HCV flare. In contrast, resolved, RNA-negative HCV does not recur, and only reinfection would account for new RNA positivity [7]. Accordingly, screening for HBV and HCV and routine patient monitoring during treatment with novel anticancer therapies are recommended [6,7]. However, there is insufficient guidance regarding HBV reactivation or HCV flares in cancer patients who receive newer anticancer drugs, including ICIs. Data on the use of combined DAAs for hepatitis C administered alongside chemotherapy and immunotherapy remain extremely limited.

## Case presentation

**Figure 1:**
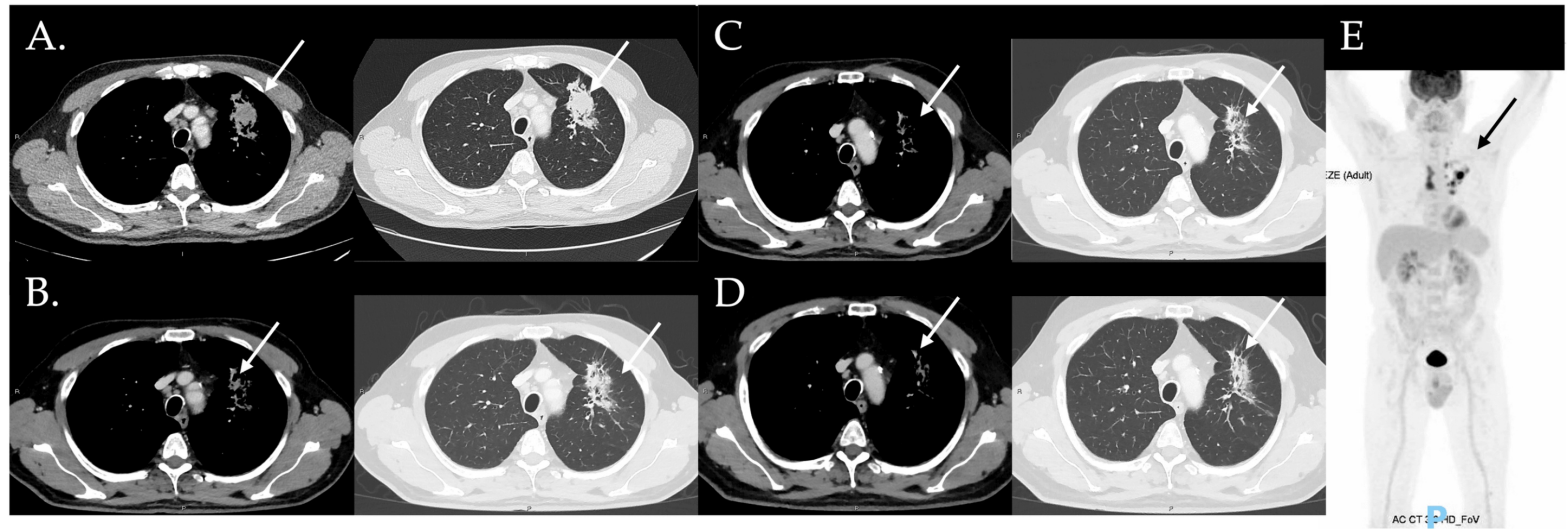
Imaging at diagnosis and follow-up Panels A-D: Thoracic CT scan performed at diagnosis and at six, eight, and 20 months of follow-up, respectively, highlighting the left upper lobe lesion (white arrow). Panel E: Fludeoxyglucose F18 injection PET scan with CT, with no evidence of liver metastasis at the time of cancer diagnosis CT: computed tomography; PET: positron emission tomography

A 56-year-old man, an active smoker with a 40-pack-year smoking history and no prior medical conditions, was diagnosed with stage IV A lung adenocarcinoma. Imaging revealed a primary lesion in the left upper lobe (67 × 32 mm), multiple infracentimetric nodules in the contralateral lung, and mediastinal and left supraclavicular lymphadenopathies (Figure 1). No sensitizing mutations were identified on broad molecular profiling performed using next-generation sequencing. PD-L1 expression was assessed using the PD-L1 IHC Dako 22C3 assay (BenchMark Ultra platform) on formalin-fixed tumor samples obtained via transbronchial biopsy. Expression was categorized according to the tumor proportion score, defined as the percentage of tumor cells showing membranous PD-L1 staining, and was estimated at 20-30%. According to current guidelines, the proposed treatment consisted of four cycles of carboplatin, pemetrexed, and pembrolizumab administered intravenously every three weeks, followed by maintenance therapy with pemetrexed and pembrolizumab every three weeks. Premedication consisted of folic acid, vitamin B12, and dexamethasone administered for three days starting the day before pemetrexed.

Baseline liver enzymes were as follows: aspartate aminotransferase (AST) 83 IU/mL (upper limit of normal (ULN) <34 IU/mL); ALT 196 IU/mL (ULN 49 IU/mL); alkaline phosphatase (ALP) 49 IU/mL (ULN <117 IU/mL); and gamma-glutamyl transferase (GGT) 168 IU/mL (ULN <73 IU/mL). Routine pretreatment viral hepatitis screening revealed positivity for anti-HCV antibodies. The first cycle of chemotherapy with carboplatin and pemetrexed, in combination with pembrolizumab, was nevertheless administered, and the patient was promptly referred to our Infectious Diseases Unit, a reference center for the treatment of viral hepatitis. Three weeks after the first cycle of chemotherapy, coinciding with the initial Infectious Diseases consultation, a significant rise in liver enzymes was observed, at which time the hepatitis C viral load was markedly elevated (1,650,000 IU/mL [6.22 log]), with genotype 4a/c/d. Regrettably, the pretreatment HCV viral load had not been assessed. Screening for HIV was negative. In retrospect, the patient recalled two to three episodes of intravenous drug use more than 30 years earlier, after which no screening for hepatitis or HIV had been performed.

While liver elastography was not performed, lung cancer staging with CT and FDG PET/CT showed no hepatic abnormalities, namely no liver metastases and no findings suggestive of advanced fibrosis or cirrhosis. Baseline noninvasive tests for liver fibrosis, such as the AST to Platelet Ratio Index (APRI) and Fibrosis-4 Index (FIB-4), were 1.226 and 1.35, respectively. These values fall below commonly used thresholds for advanced fibrosis (APRI >1.5; FIB-4 >3.25) and are therefore consistent with a low to intermediate risk of fibrosis. Alpha-fetoprotein (AFP) was 22.9 IU/mL (ULN <8.78 ng/mL). Given active hepatitis with elevated transaminases, the absence of focal hepatic lesions on CT or PET/CT, and the lack of cirrhotic features, this modest elevation in AFP was considered nonspecific, and a paraneoplastic contribution from lung adenocarcinoma could not be excluded. 

At the time of the initial consultation at the Infectious Diseases Unit, combination G/P was promptly initiated after confirming the absence of known drug interactions with the ongoing oncologic treatment. Direct-acting antivirals were continued for eight weeks, during which liver enzyme levels progressively decreased, allowing for completion of the planned chemotherapy and immunotherapy schedule administered every three weeks. HCV treatment was very well tolerated, and there was no need for treatment interruption or dose adjustment throughout this period. At week four of G/P therapy, liver enzymes had normalized, and the HCV viral load was undetectable, both at this time point and at the end of treatment. Sustained virologic response was confirmed at 12, 24, and 48 weeks after treatment completion. 

After the first four cycles of cancer treatment, the CT scan revealed a favorable partial response; carboplatin was discontinued according to protocol, and the patient continued therapy with pemetrexed and pembrolizumab. A subsequent whole-body FDG PET/CT scan showed a favorable dimensional and metabolic response of all neoplastic lesions (Figure 1). A mild increase in transaminases was first noted after the seventh cycle. Both pemetrexed and pembrolizumab were continued for an additional 11 cycles, after which ALT reached 2.5 times the ULN (Common Terminology Criteria for Adverse Events (CTCAE) version 5.0, Grade 1 [8]). Pembrolizumab was withheld at the subsequent cycle, but transaminase levels did not normalize.

After the 19th combined treatment cycle, persistent mild elevation of hepatic enzymes was again observed. Pemetrexed was subsequently reintroduced as monotherapy, after which AST peaked at approximately 4.47 times the ULN and ALT at 4.14 times the ULN (both CTCAE version 5.0, Grade 2) 13 days later. Transaminase levels normalized following permanent discontinuation of pemetrexed, and pembrolizumab monotherapy was resumed 36 days later without recurrent hepatotoxicity. Taken together, these findings support pemetrexed as the most likely cause of the transaminitis, as there was no evidence of immune-related hepatitis attributable to checkpoint inhibition, nor any evidence of HCV reactivation.

**Figure 2:**
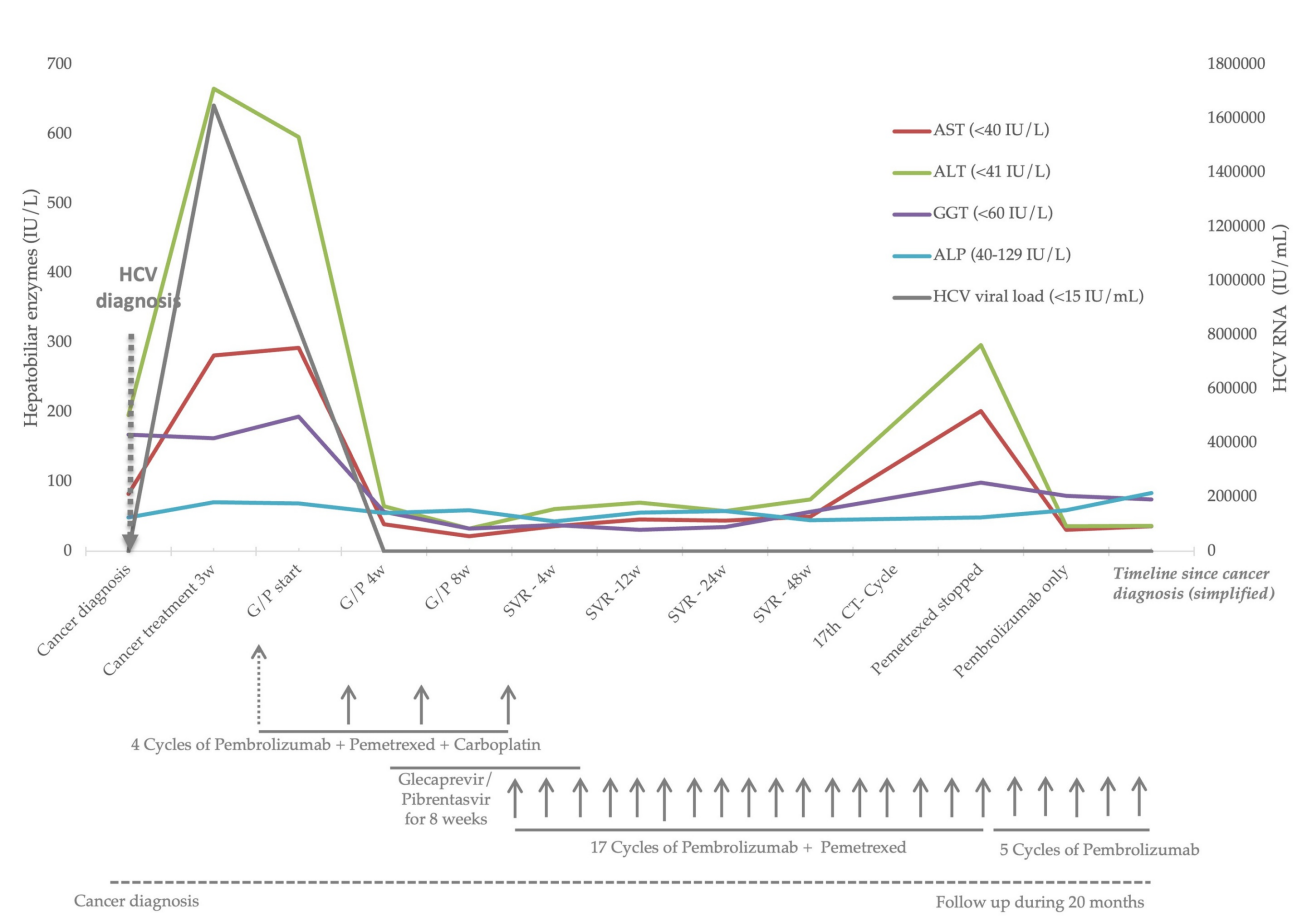
Liver enzyme and HCV viremia evolution during chemo/imnumotherapy and hepatitis C treatment The figure shows liver enzymes in IU/L (in parentheses, the upper limit of normal), as well as HCV viral load. Below, each arrow represents a chemo/immunotherapy cycle. Routine viral hepatitis pre-screening revealed antibodies to anti-hepatitis C before the start of cancer treatment. The first cycle of chemotherapy with carboplatin and pemetrexed together with pembrolizumab was nevertheless administered the following day. Three weeks later, there was a significant rise in liver enzymes. The patient was immediately started on G/P for eight weeks, resulting in progressive normalization of liver enzymes, allowing for the completion of the proposed combination chemotherapy with immunotherapy – another three cycles every three weeks. Treatment was continued with pembrolizumab and pemetrexed until the latter was stopped, due to changes in liver enzymes attributable to this drug. So far, the patient has been treated with 26 cycles, with a favorable oncological response ALT: alanine transaminase; AST: aspartate transaminase; CT: cancer treatment; GGT: gamma-glutamyl transpeptidase; G/P: glecaprevir/pibrentasvir; HCV: hepatitis C virus; SVR: sustained virological response; w: weeks

Treatment remains ongoing, with 26 cycles of oncologic therapy completed, and a favorable oncologic response. Details of pre-treatment and subsequent HCV viremia, liver enzyme levels, and treatment schedules are summarized in Table 1 and Figure 2.

**Table 1:** Laboratory findings during follow-up period ALP: alkaline phosphatase; ALT: alanine transaminase; AST: aspartate transaminase; CT: cancer treatment; NA: not assessed; n.d.: non-detectable; GGT: gamma-glutamyl transferase; G/P: glecaprevir/pibrentasvir; HCV: hepatitis C virus; SVR: sustained virological response; w: weeks

	HCV viral load (<15 IU/mL)	AST (<40 IU/L)	ALT (<41 IU/L)	GGT (<60 IU/L)	ALP (40-129 IU/L)
Cancer diagnosis	NA	83	196	168	49
Cancer treatment 3w	1650000	282	666	163	71
G/P start		293	596	194	69
G/P 4w	n.d.	39	65	57	55
G/P 8w	n.d.	22	33	33	59
SVR - 4w	n.d.	36	61	38	43
SVR -12w	n.d.	46	70	31	56
SVR - 24w	n.d.	44	58	35	58
SVR - 48w	n.d.	50	75	57	45
17th CT- Cycle	n.d.	202	297	99	49

Of note, at initial screening before hepatitis C treatment, anti-hepatitis A virus antibodies were negative, and hepatitis B serology showed negative HBs antigen, undetectable anti-HBs antibodies, positive anti-HBc antibodies, negative anti-HBe antibodies, and HBe antigen, as well as an undetectable HBV viral load. As isolated anti-HBc positivity may reflect past infection, occult HBV infection, or false positivity, a cautious approach was adopted in the immunosuppressed setting. The patient received hepatitis A vaccination for prevention and one dose of hepatitis B vaccine, after which anti-HBs antibodies were 7 IU/mL. Subsequently, after completion of a primary three-dose immunization schedule, the anti-HBs titer increased to 867 IU/mL. Given an adequate response to HBV vaccination and isolated anti-HBc positivity in the presence of negative HBe antigen and anti-HBe antibodies, this profile was considered more consistent with false positivity rather than prior HBV infection. No HBV reactivation was detected during follow-up.

## Discussion

While the former standard of care for HCV treatment with ribavirin and pegylated interferon posed substantial challenges, current DAAs are safe and well-tolerated. Nonetheless, potential drug-drug interactions must be anticipated to avoid toxicity. In this case, possible drug-drug interactions were reviewed using the UpToDate and Liverpool Hep Drug Interaction databases, with particular attention to CYP3A, P-gp, and OATP pathways relevant to glecaprevir/pibrentasvir. No clinically meaningful interactions were identified with carboplatin, pemetrexed, or pembrolizumab.

In 2016, a prospective observational study analyzed 21 HCV-infected cancer patients: 12 solid tumors (breast, hepatocellular, cholangiocarcinoma, esophageal, colon, pharyngeal) and nine hematologic malignancies (multiple myeloma, myelodysplastic syndrome, acute myelogenous leukemia, diffuse large B-cell lymphoma, follicular lymphoma, Waldenström macroglobulinemia, mycosis fungoides/T-cell lymphoma) [9]. Patients received concomitant chemotherapy and HCV treatment with sofosbuvir-containing regimens (sofosbuvir-ledipasvir±ribavirin, sofosbuvir±ribavirin, sofosbuvir-simeprevir, or sofosbuvir-daclatasvir) per contemporaneous recommendations [9]. During concomitant anti-cancer and HCV treatment, for a median duration of 82 days, the most common adverse events (AEs) were constitutional (12; 57%), hematological and gastrointestinal (7; 33%) in nature. When present, serious AEs (Grade 3-4) occurred within two to four weeks of initiation of concomitant DAAs and chemotherapy. Serious AEs such as Grade 3 AEs occurred in eight patients and were mostly hematological. Despite occasional ribavirin dose reduction or chemotherapy modification, no treatments were discontinued, and SVR was 95% (20/21) [7], supporting the safety of concomitant chemotherapy and DAAs in cancer patients with chronic HCV.

A pivotal 2019 prospective observational study with 153 patients showed efficacy and safety of sofosbuvir-based regimens in cancer patients with HCV infection. Of the 32 patients initially excluded from cancer clinical trials because of HCV, 27 (84%) were granted cancer therapy access after starting DAA treatment [10]. A 2020 systematic review that included 186 patients from 14 studies focused on the safety and efficacy of ICIs in HBV or/and HCV-infected cancer patients, of which 43% were undergoing antiviral treatment [11]. Hepatic transaminase elevation occurred in 22% of HBV or HCV-infected patients, of which 10.8% were Grade 3 or 4, reversible by antiviral therapy or corticosteroids, without ICIs interruption, although it is not completely clear if the included patients had ongoing active viral hepatitis [11].

Other reports provide similar evidence regarding the safety and efficacy of ICIs in patients with chronic HCV infection [12,13]. In 2022, a prospective observational study evaluated 47 cancer patients with HCV infection treated with either G/P for eight weeks or ledipasvir/sofosbuvir for 12 weeks, of whom 17 patients received concomitant oncologic therapy. There were no significant differences between the two regimens, with an overall SVR of 96% (45/47; 95% CI: 86-99%), supporting the efficacy and safety of DAAs, including short eight-week regimens, in cancer patients with HCV infection [12]. In our reported clinical case, we selected an eight-week G/P regimen after confirming the absence of relevant drug-drug interactions with ongoing cancer therapy.

HCV infection is a major cause of liver cancer and is also associated with several non-hepatic malignancies, including B-cell non-Hodgkin lymphoma, cholangiocarcinoma, and pancreatic cancer [13]. Moreover, a systematic review and meta-analysis including data from 145,614 HCV-infected patients found that chronic HCV infection was significantly associated with an increased risk of lung cancer, with a pooled relative risk of 1.94 (95% CI: 1.56-2.42; I² = 87%) [14]. It remains uncertain whether this association is causative, based on molecular and immune pathways, or reflects exposure to carcinogenic agents common to both conditions. Beyond shared exposures such as smoking, proposed mechanisms include chronic cytokine signaling and immune dysregulation (e.g., IL-6, NF-κB), systemic inflammatory and oxidative environments with impaired immune surveillance, and virus-host interactions that disrupt cell-cycle and apoptotic pathways (reported for HCV core and NS proteins in extrahepatic tissues), although direct causality in lung tissue has not been established [14,15].

Despite the clear advantages of screening for and treating hepatitis C in any clinical setting, it is worth noting that many cancer centers do not routinely screen patients for HCV. The 2012 estimated prevalence of chronic HCV infection among cancer patients in the United States, ranging from 1.5% to 10.6%, is likely an underestimate, as suggested by a 2014 observational study reporting that only 13.9% of 141,877 cancer patients were screened for HCV infection [16]. Furthermore, despite the undisputed efficacy of modern DAAs for HCV, available since 2013, cancer patients with HCV infection are still frequently excluded from oncology clinical trials. A 2017 study analyzing 5,638 registered phase 1-4 oncology clinical trials found that 58% of these trials excluded patients with chronic HCV infection [17].

In a 2018 prospective observational study, HCV-related events were reported in 23 of 100 HCV-infected cancer patients (23%) receiving chemotherapy, resulting in hepatitis flare in 10 of these 23 patients (43%). Only six of the 23 patients (26%) experiencing HCV reactivation required unplanned discontinuation or dose reduction of chemotherapy, supporting the need to identify and treat chronic HCV infection to avoid detrimental changes in cancer therapy [18]. However, as recently as 2022, HCV flare prevalence in cancer patients ranged from 1.5% to 32% worldwide, with a substantial proportion of patients, up to 31%, unaware of their viral status at cancer diagnosis. As a result, changes in oncologic therapy have been documented in up to 8% of cases, with side effects sometimes mistakenly attributed to cancer treatment rather than to viral hepatitis flare [19].

Anti-HCV antibody positivity alone does not differentiate between active and past HCV infection. To make this distinction, HCV viral load should be measured in patients with positive anti-HCV antibodies, ideally on two to three separate occasions, because HCV viremia can fluctuate, and a single negative viral load may not be sufficient to exclude active infection. Studies on HCV infection in cancer patients do not always use HCV viral load to distinguish between past and active infection. The term “HCV-related hepatitis flare” is defined as an unexplained increase in alanine aminotransferase to three times the upper limit of normal during chemotherapy, along with an increase in HCV-RNA of ≥1 log₁₀ IU/mL over baseline. In reference to our patient, although hepatitis C viral load was not measured before cancer treatment, we believe that the elevated baseline liver enzymes and their subsequent rise during oncologic therapy likely reflect an HCV-related hepatitis flare. 

Observational studies report that the use of DAAs in cancer patients with HCV infection may potentially contribute to improved liver function, reduced liver disease progression, facilitate access to clinical trials, chemotherapy, and transplantation, prevent deleterious changes in cancer treatment plans, and support improved patient outcomes [17]. Our single case report illustrates the feasibility of this approach, demonstrating biochemical and virologic response, but it does not allow outcomes to be established. Whenever cancer treatment should not be withheld or interrupted, concomitant DAAs and cancer treatment may be performed with close monitoring, particularly during the first four weeks of concomitant treatment, since serious adverse events are more frequent within the first two to four weeks of therapy [19]. HCV treatment with DAAs should be started in patients with cancer and survivors without significant delay, provided there are no contraindications, potential drug-drug interactions, or overlapping toxicities with anti-neoplastic drugs. DAAs may be initiated before, during, or after cancer treatment [20]. In our patient, our management approach was consistent with these recommendations.

Lastly, another important aspect to consider regarding HCV treatment in cancer patients is the theoretical risk of HBV reactivation in HBV/HCV co-infected patients [20]. In our reported clinical case, HBV serology showed mono-positivity for anti-HBc antibodies, undetectable HBsAg and HBeAg, negative anti-HBs and anti-HBe antibodies, and an undetectable HBV viral load. This serologic profile, according to recommendations, is usually interpreted as evidence of prior exposure to the HBV virus, and therefore, the patient would not benefit from vaccination to prevent HBV infection. According to international guidelines from the European Association for the Study of the Liver (EASL) and the American Association for the Study of Liver Diseases (AASLD), depending on clinical circumstances and the feasibility of close monitoring, individuals undergoing cancer treatment or other immunosuppressive therapies who present with mono-anti-HBc antibody positivity could either receive anti-HBV prophylaxis or be closely monitored, with on-demand anti-HBV therapy initiated at the first sign of HBV reactivation [6].

Although isolated anti-HBc individuals may adequately produce anti-HBs after vaccination or a single booster, which could indicate prior exposure, vaccine response alone cannot distinguish false-positive anti-HBc from resolved or occult infection. In immunosuppressed patients, serial HBsAg and HBV DNA monitoring, along with prophylaxis or preemptive therapy when indicated, remains the cornerstone of management. Nonetheless, the observed response to HBV vaccination in our patient with mono-anti-HBc positivity suggests no prior HBV exposure and further highlights the need for improved biomarkers to assess previous exposure, the requirement for vaccination, and expected vaccine immunogenicity, particularly in individuals undergoing immunosuppressive treatments.

## Conclusions

This clinical case illustrates how recent DAA drugs for HCV treatment have enabled successful therapy in various at-risk and difficult-to-treat populations, including non-HIV immunocompromised individuals and cancer patients. In this case, rapid initiation of HCV treatment allowed cancer therapy to continue without unnecessary interruption. In this context, both cancer and HCV progression posed risks, and timely HCV diagnosis and treatment enabled the continuation of cancer therapy while preventing liver disease progression. More specifically, we provide practical insights into the management of chronic hepatitis C in cancer patients, further emphasizing the importance of routine viral infection screening before the initiation of cancer treatment. Our report also demonstrates the feasibility and value of HCV treatment and its positive clinical outcomes. Prompt diagnosis and treatment of viral infection prevented liver disease progression and allowed cancer therapy to proceed without significant adverse events or drug-drug interactions, contributing to overall treatment success and quality of life. Additional research could inform specific guidelines and facilitate the inclusion of HCV-positive patients in oncology clinical trials.
